# A Long-Term (41 Years) Radiographic Follow-Up Study of the Onset and Development of a Jawbone Lesion in a Patient With Gnathodiaphyseal Dysplasia

**DOI:** 10.7759/cureus.88763

**Published:** 2025-07-25

**Authors:** Riyu Koguchi, Haruhisa Watanabe, Mai Nishiura, Tadahiro Iimura, Yutaka Maruoka

**Affiliations:** 1 Department of Oral Diagnosis and Medicine, Hokkaido University, Faculty of Dental Medicine/Graduate School of Dental Medicine, Sapporo, JPN; 2 Department of Pharmacology, Hokkaido University, Faculty of Dental Medicine/Graduate School of Dental Medicine, Sapporo, JPN; 3 Department of Dentistry for Children and Disabled Persons, Hokkaido University, Faculty of Dental Medicine/Graduate School of Dental Medicine, Sapporo, JPN; 4 Department of Oral Surgery, National Center for Global Health and Medicine, Japan Institute for Health Security, Tokyo, JPN

**Keywords:** autosomal dominant genetic disorder, bone metabolism, cemento-osseous lesion, gnathodiaphyseal dysplasia (gdd), jaw bone

## Abstract

Gnathodiaphyseal dysplasia (GDD) is an autosomal dominant syndrome characterized by bone fragility, sclerosis of tubular bones, and cemento-osseous lesions of the jawbones. This report describes the long-term radiographic follow-up study of jaw lesions in a GDD-affected woman of >40 years of age from the age of one onward. At three years of age, widening of the diaphyseal cortices in the femur and radius was observed, although no symptoms were observed in the jawbones. At nine years of age, we observed a slightly sclerotic appearance in the alveolar and jawbones adjacent to the permanent tooth roots. After completion of the secondary dentition at 14 years of age, increased density of the sclerotic mass was clearly observed. Although the etiology and pathogenesis are uncertain, this study observed that the onset of the jawbone lesion had already appeared after the mixed dentition stage, and the sclerotic mass developed with age.

## Introduction

In 1969, Akasaka et al. studied a large four-generation family including 21 patients with frequent bone fragility at a young age and purulent osteomyelitis of the jaws during adult life, with autosomal dominant inheritance [1]. In this family, the patients experienced frequent bone fractures caused by trivial occurrences; however, the fractures healed normally without any bone deformity. Radiographic examinations revealed multiple radiopaque lesions in the upper and lower jaws, and bilateral thickening of the diaphyseal cortices of the long bones. Histological examination of such jaw lesions is compatible with cementoosseous dysplasia. This syndrome was distinguished from known systemic bone diseases as a rare clinical entity characterized by bone fragility, long-bone sclerosis, and cemento-osseous lesions of jawbones with autosomal dominant inheritance, with high penetrance, and was named “gnathodiaphyseal sclerosis.”

In 1985, Levin et al. described other families sharing many features with Akasaka’s entity under the name of “osteogenesis imperfecta with unusual skeletal lesions (MIM166260)” [2]. Sporadic cases with similar clinical manifestations have been reported. In 2001, Riminucci et al. proposed “gnathodiaphyseal dysplasia (GDD)” as the new name for this disease entity instead of “gnathodiaphyseal sclerosis” because osteosclerosis is not a feature of the jaw lesions of these patients [3]. Based on a linkage analysis of this family, we mapped the GDD locus to an 8.7-cM interval on chromosome 11p14.3-15.1 and identified a novel gene (GDD1) responsible for this syndrome by a positional cloning analysis [4]. GDD1 cDNA encodes a 913-amino-acid protein containing eight putative transmembrane-spanning domains [5,6]. Missense mutations occurred at the cysteine residue of amino acid 356 in this family.

We clinically investigated this family through five generations, including 12 males and 14 females. Among them, we focused on a GDD-affected woman of >41 years of age from one year of age. This case report focuses on GDD and describes the long-term (41 years) follow-up of clinical and radiographic examinations. Although the etiology and pathogenesis of cemento-osseous dysplasia are still uncertain, this study clearly illustrates the onset of jaw lesions that appeared after mixed dentition, and that the sclerotic mass matured with the growth process. This is a report that involves a subsequent study on the origin of cemento-osseous dysplasia in GDD.

## Case presentation

**Figure 1:**
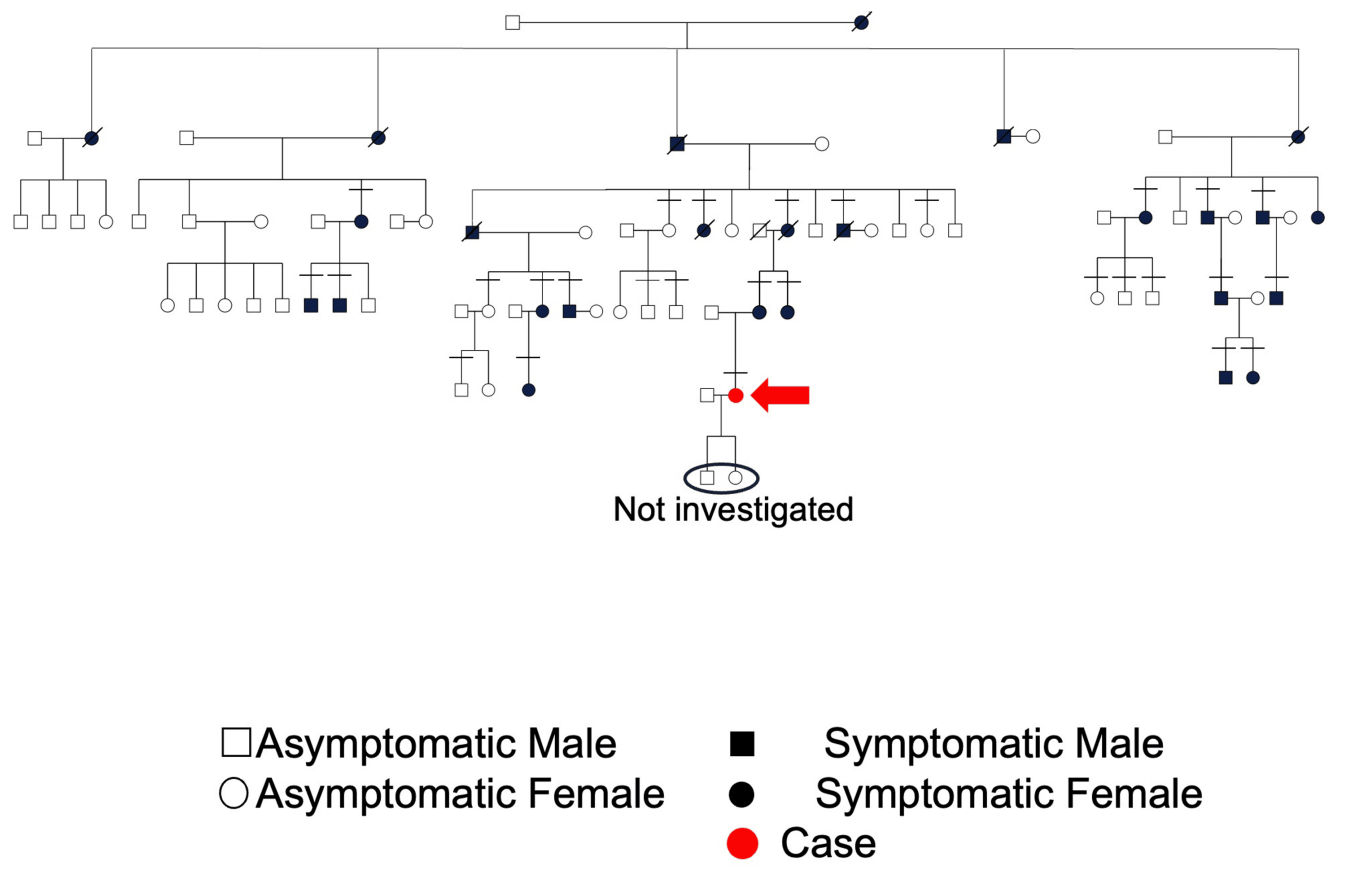
Family tree of a GDD-affected family in Japan The red arrow indicates a GDD1-affected female whose clinical features are described in this paper GDD: gnathodiaphyseal dysplasia

We clinically investigated a family genetically diagnosed with GDD, involving five generations of family members aged 19-89 years, including 12 males and 14 females (Figure 1, case marked by horizontal bars in pedigree). All family members with GDD had normal stature, sclerae, hearing, and teeth, without appealing orthopedic symptoms, such as bone fractures and joint pain. Moreover, there was no evidence of café-au-lait spots on the skin or endocrine organ tumors. Among them, we clinically followed up a GDD1-affected female in this family from the age of one (Figure 1).

**Figure 2:**
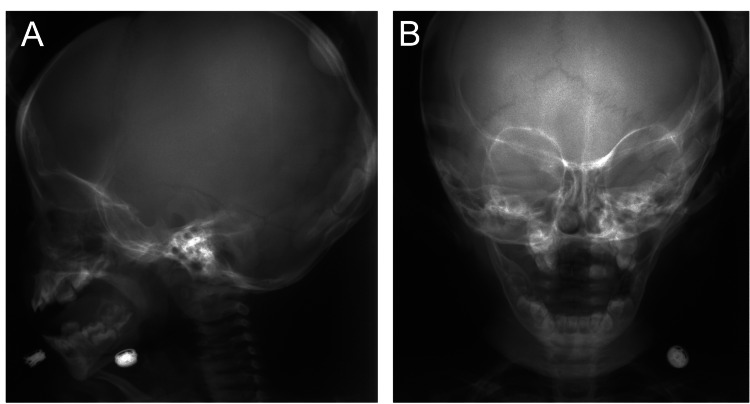
Skull radiograph at age one A: Lateral view. B: Frontal view. No clinical or radiographic findings are observed in the maxillofacial region

**Figure 3:**
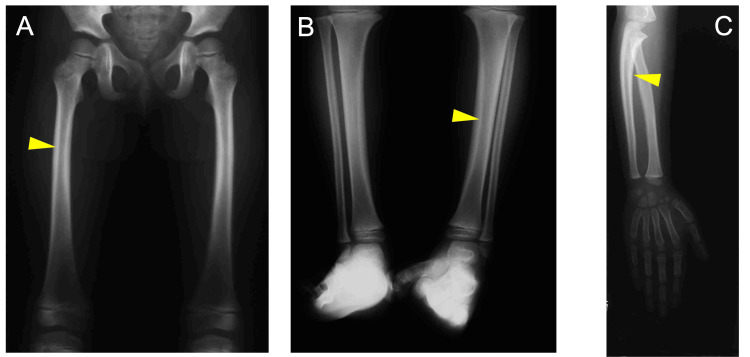
Radiographs of long bones at age three A: Femur. B: Tibia. C: Ulna. Yellow arrowheads indicate thickening of the cortices of limb bones

**Figure 4:**
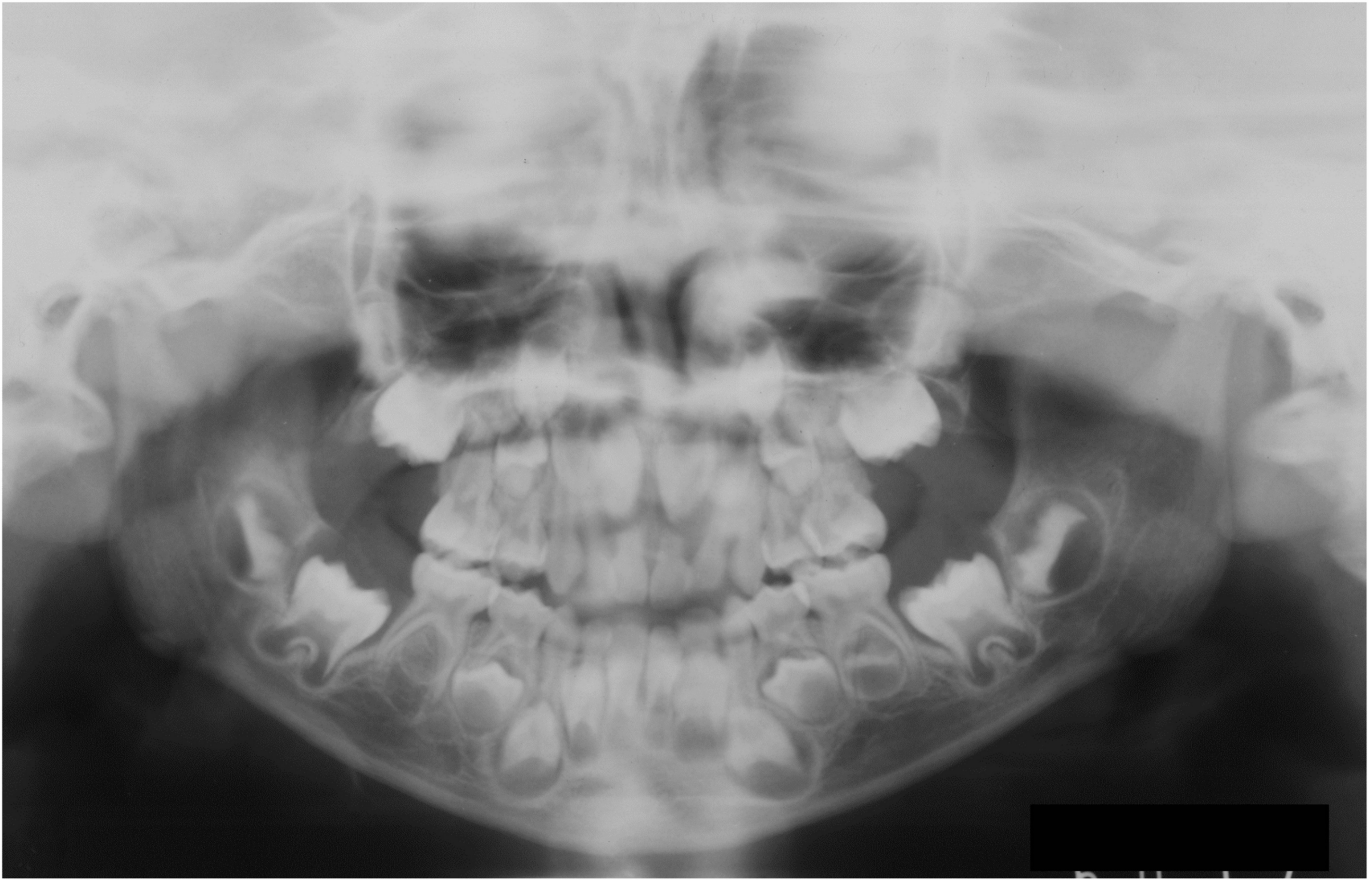
Maxillofacial radiograph at age six No abnormal findings in the maxillofacial bones are observed

At the age of one, no clinical or radiographic findings were observed in the maxillofacial region (Figure 2). At three years of age, we observed thickening of the cortical bone of the femurs, tibiae, and ulnae (yellow arrowheads), which is a typical symptomatic appearance observed in the long bones of patients with GDD (Figure 3). We did not observe any abnormal findings in the maxillofacial bones until six years of age, which corresponds to Hellmann's dental age IIC (Figure 4). These findings indicate that the appearance of a long bone phenotype preceded that of the jawbone phenotype in a patient with GDD.

**Figure 5:**
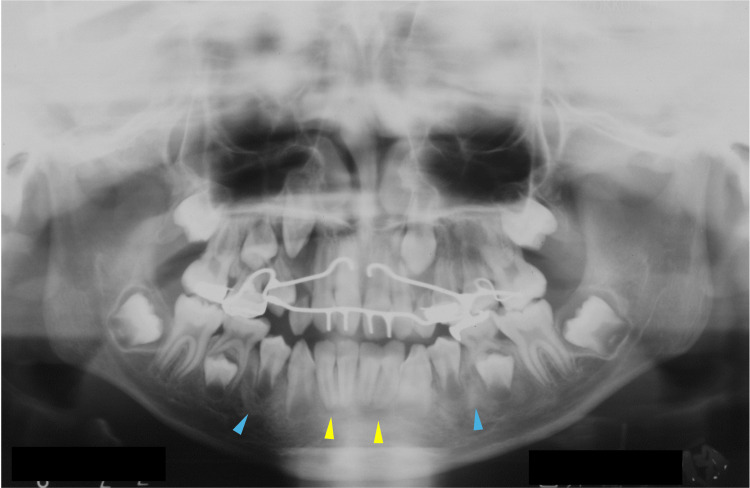
Maxillofacial radiograph at age nine Blue arrowheads indicate slight hardening of the bone lesions adjacent to the roots of the permanent mandibular teeth. Yellow arrowheads indicate a radiolucent area at the apical site of the lower frontal teeth

**Figure 6:**
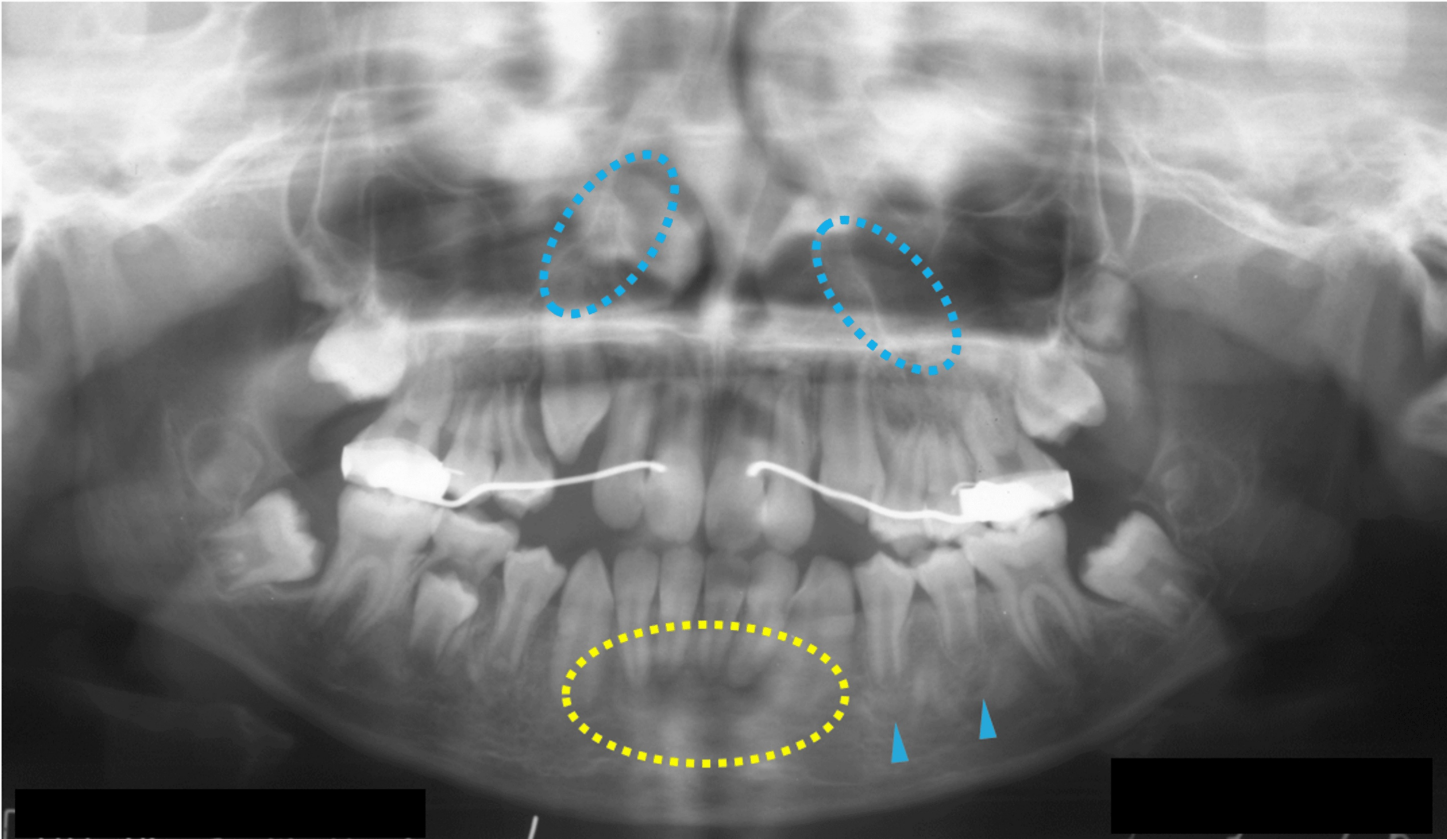
Maxillofacial radiograph at age 10 Blue dotted circles indicate a cotton-like radiopaque image of cemento-osseous lesions in the maxillary sinus. Blue arrowheads indicate opacities in the mandible bone areas encircling the roots of the premolar teeth. Yellow dotted circles indicate a radiolucent lesion in the mandibular bone area apical to the incisors

At nine years of age, corresponding to Hellmann's dental age IIIB, the patient began to receive orthodontic treatment for aesthetic reasons. Even though an orthodontic apparatus was used for the maxilla, slight hardening of the bone lesions was observed adjacent to the roots of the permanent mandibular teeth (Figure 5, blue arrowheads). In addition, a radiolucent area was observed at the apical site of the lower frontal teeth (Figure 5, yellow arrowheads). At 10 years of age, bone lesions became obvious and were observed at multiple sites of the craniofacial bones (Figure 6). A cotton-like radiopaque image, the typical radiographic appearance of cemento-osseous lesions, was observed in the maxillary sinus (Figure 6, blue dotted circles). Opacities in the mandible were observed in the bone areas encircling the roots of the premolar teeth (Figure 6, blue arrowheads). A radiolucent lesion was also observed in the bone area apical to the mandibular incisors (Figure 6, yellow dotted circles).

**Figure 7:**
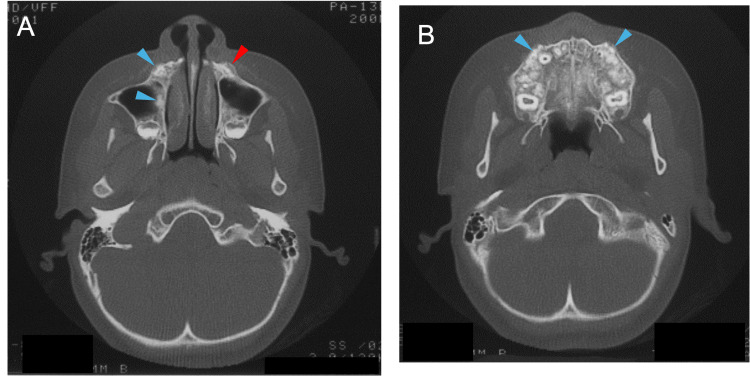
Horizontal CT images illustrating jawbone lesions at age 11 A: Maxillary sinus. B: Maxillary alveolar bone. Blue arrowheads indicate irregularly marginated sclerotic calcified lesions in the walls of the maxillary sinus (the left panel) and the front walls surrounding the canine (the right panel), respectively. The red arrowhead indicates a periosteal reaction in the left maxilla CT: computed tomography

A close examination by CT at 11 years of age revealed irregularly marginated sclerotic calcified lesions in the walls of the maxillary sinus (Figure 7, blue arrowheads in the left panel) and the front walls surrounding the canine (Figure 7, blue arrowheads in the right panel). The lesions in the back and front walls appeared to be confined to the trabecular bone and periapical areas of the canines, respectively, but did not erode into the cortical bone. A periosteal reaction was also observed in the left maxilla (Figure 7, red arrowhead). These findings indicated that bone lesions in the jawbones started to be observed and increased in number in the mixed dentition stages.

**Figure 8:**
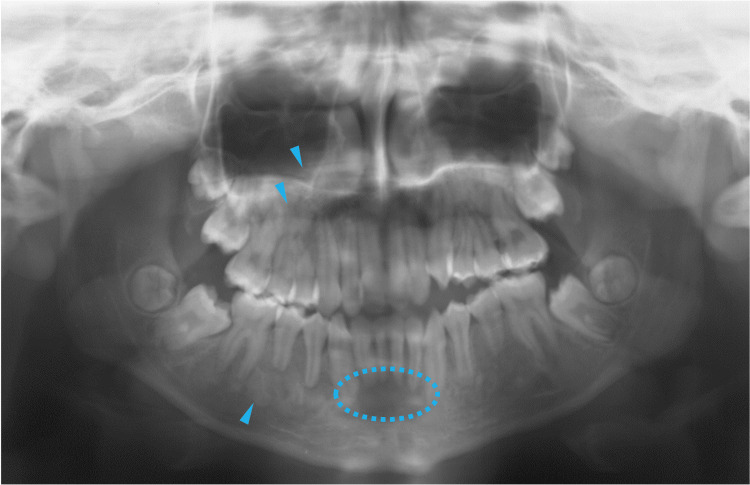
Maxillofacial radiograph at age 12 Blue arrowheads indicate the opacities in the maxillary sinus and mandibular premolar regions. The blue dotted circle indicates the opacity in the anterior mandible region

**Figure 9:**
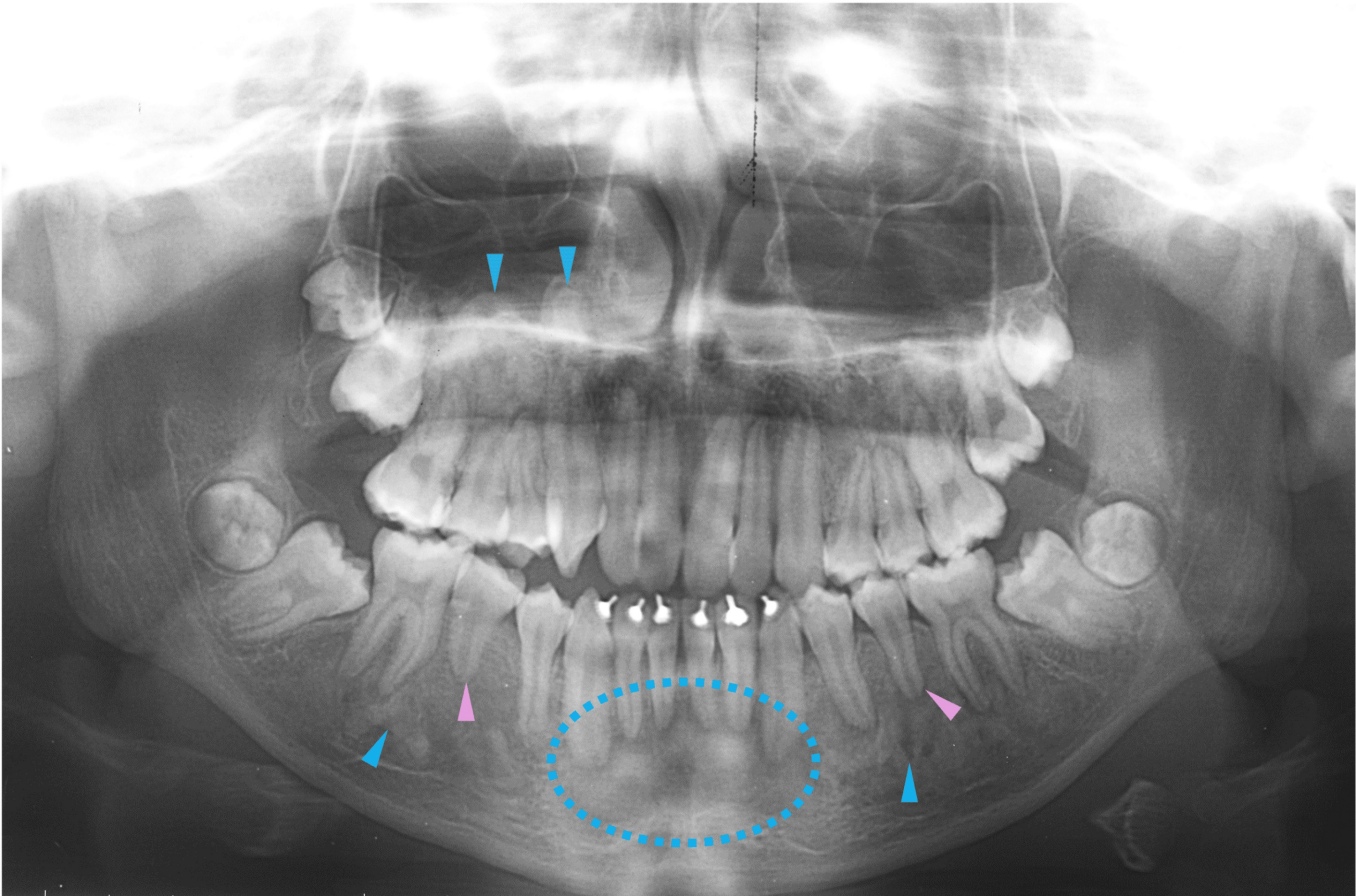
Maxillofacial radiograph at age 13 Blue arrowheads indicate the opacities in the maxillary sinus and new sclerotic lesions at the apical sites of the mandibular premolars. The blue dotted circle indicates the sclerotic lesion in the anterior mandibular region. Purple arrowheads indicate the short roots of the second molars

At 12 years of age, the opacities in the maxillary sinus and mandibular premolar regions intensified (Figure 8, blue arrowheads), while the opacity in the anterior mandible region decreased (Figure 8, blue dotted circle), suggesting active tissue displacement or remodeling of bone lesions. One year later, at 13 years of age, the opacities in the maxillary sinus were further intensified (Figure 9, blue arrowheads). Notably, the lesion in the anterior mandibular region became sclerotic (Figure 9, blue dotted circle), and new sclerotic lesions were observed at the apical sites of the mandibular premolars (Figure 9, blue arrowheads). These new sclerotic lesions appeared to be associated with the short roots of the second molars (Figure 9, purple arrowheads). These findings indicated that the bone lesions in the jawbones showed active tissue remodeling and increased in number and size in the late mixed dentition stages.

**Figure 10:**
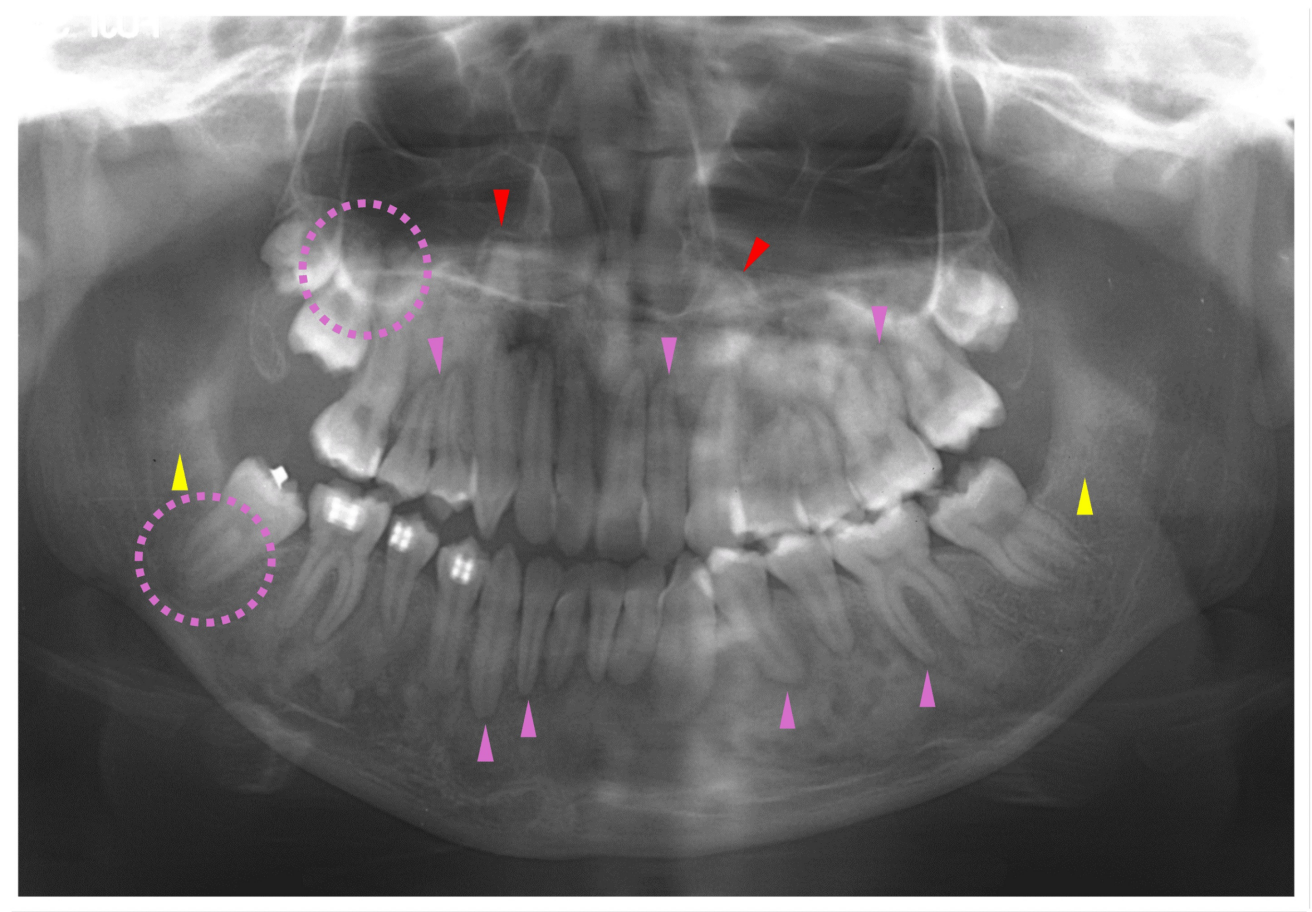
Maxillofacial radiograph at age 14 Yellow arrowheads indicate normal healings of the mandibular bone sites after the impacted wisdom teeth extraction. Purple dotted circles indicate bony adhesions (ankylosis) in the right upper and lower second molars. Purple arrowheads indicate widening of the periodontal ligament spaces. Red arrowheads indicate stenosis of the maxillary sinus

At 14 years of age, stage IVA in Hellman's dental age, which was the completion stage of secondary dentition, impacted mandibular wisdom teeth were extracted for orthodontic reasons and appeared to heal normally without infection (Figure 10, yellow arrowheads). The opacities of sclerotic lesions in the mandibular molar regions intensified, and these lesions expanded to fuse with each other (Figure 10). The right lower second molar showed bony adhesion (ankylosis) (Figure 10, purple dotted circle). Similarly, another bony adhesion (ankylosis) was observed in the right upper second molar (Figure 10, purple dotted circle). Widening of the periodontal ligament spaces was observed in multiple teeth (Figure 10, purple arrowheads). Stenosis of the maxillary sinus was observed bilaterally (Figure 10, red arrowheads).

**Figure 11:**
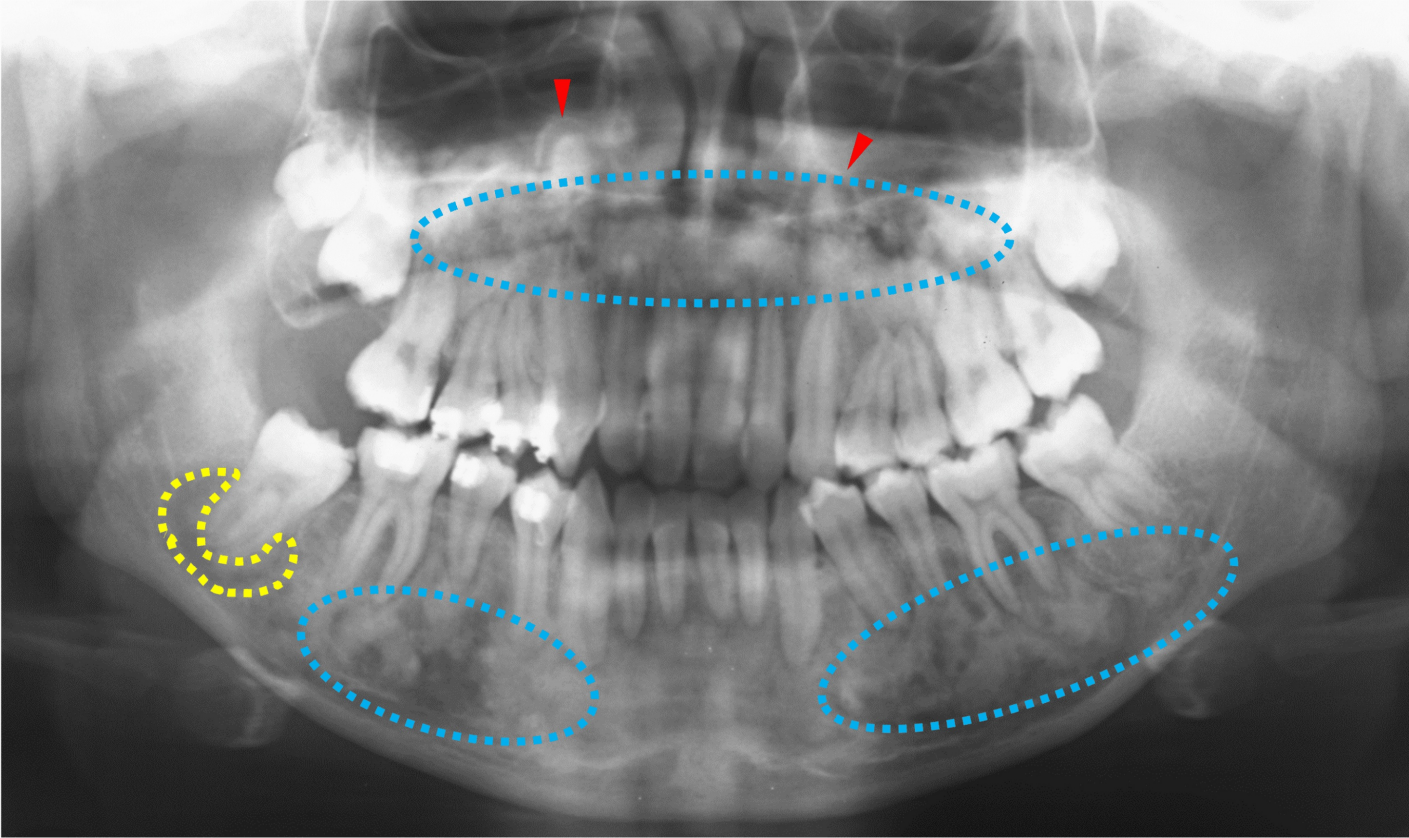
Maxillofacial radiograph at age 15 Blue dotted circles indicate the lesions in both the upper and lower jaws with enlarged frosted shadows. Red arrows indicate stenosis of the maxillary sinus. The yellow dotted circle indicates a radiolucent lesion adjacent to the right mandibular second molar root

**Figure 12:**
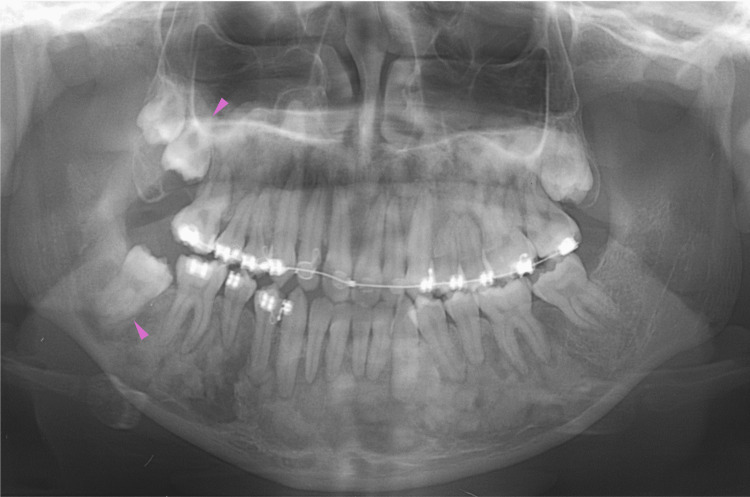
Maxillofacial radiograph at age 17 Purple arrowheads indicate bony adhesions (ankylosis) in the right upper and lower second molars

**Figure 13:**
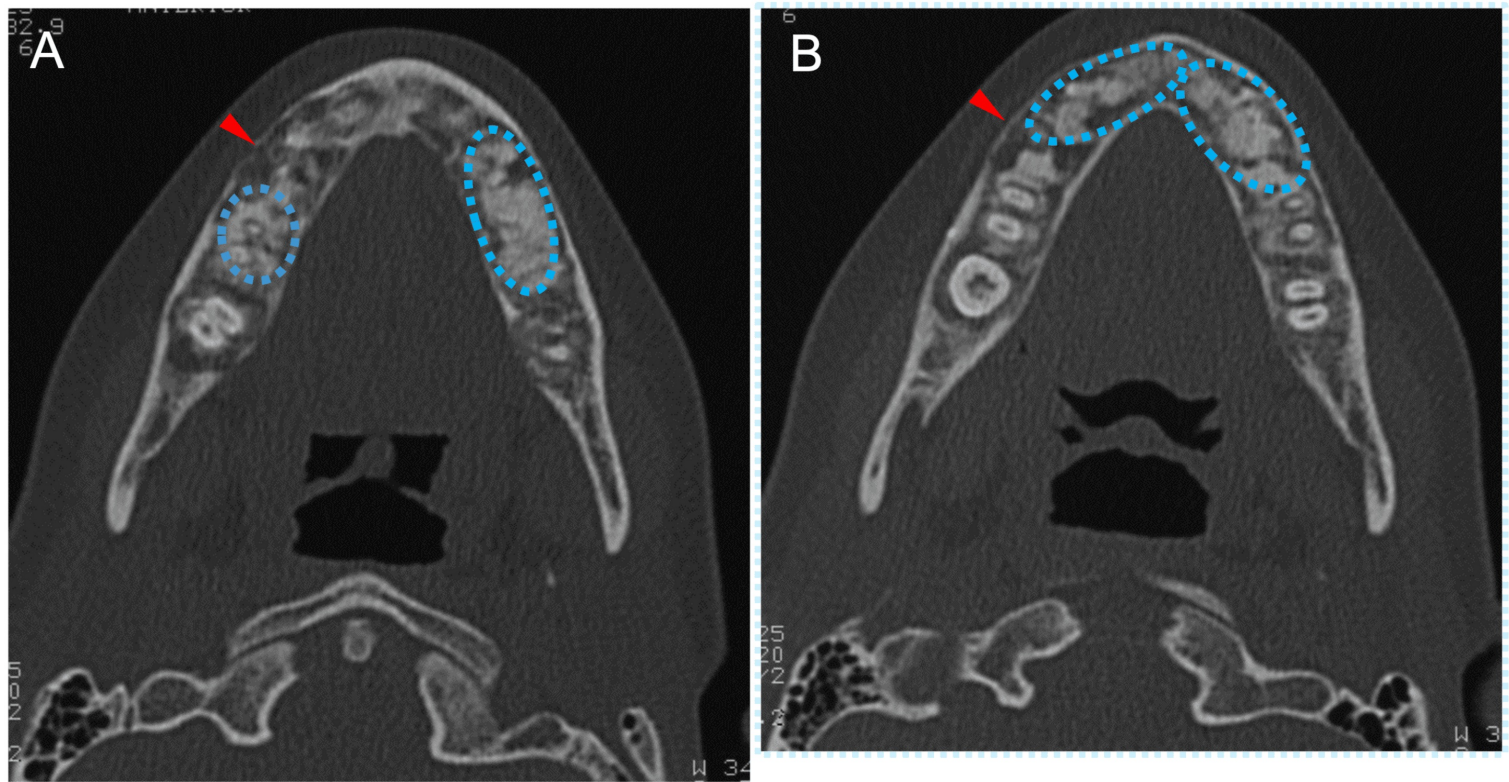
Horizontal CT images illustrating jawbone lesions at 19 years of age A, B: Lower jaw, slightly distinct horizontal slice levels. Red arrowheads indicate eroded cortical bone by lesions. Blue dotted circles indicate irregularly margined sclerotic calcified lesions CT: computed tomography

At 15 years of age, the lesions in both the upper and lower jaws showed enlarged frosted shadows (Figure 11, blue dotted circles), of which the upper lesions were associated with stenosis of the maxillary sinus (Figure 11, red arrowheads). In addition, a lesion adjacent to the right mandibular second molar root was radiolucent (Figure 11, yellow dotted circle) and became a bony adhesion (ankylosis) at 17 years of age (Figure 12, purple arrowhead). Similarly, another bony adhesion (ankylosis) was observed in the right upper second molar (Figure 12, purple arrowhead). Two years later, at 19 years of age, CT images of the lower jaw showed eroded cortical bone by lesions (Figure 13, red arrowheads) as well as irregularly margined sclerotic calcified lesions (Figure 13, blue dotted circles). These findings indicated that the bone lesions in the jawbones grew further, affecting the cortical bone and periodontal tissue in the completion stage of secondary dentition and later stages.

**Figure 14:**
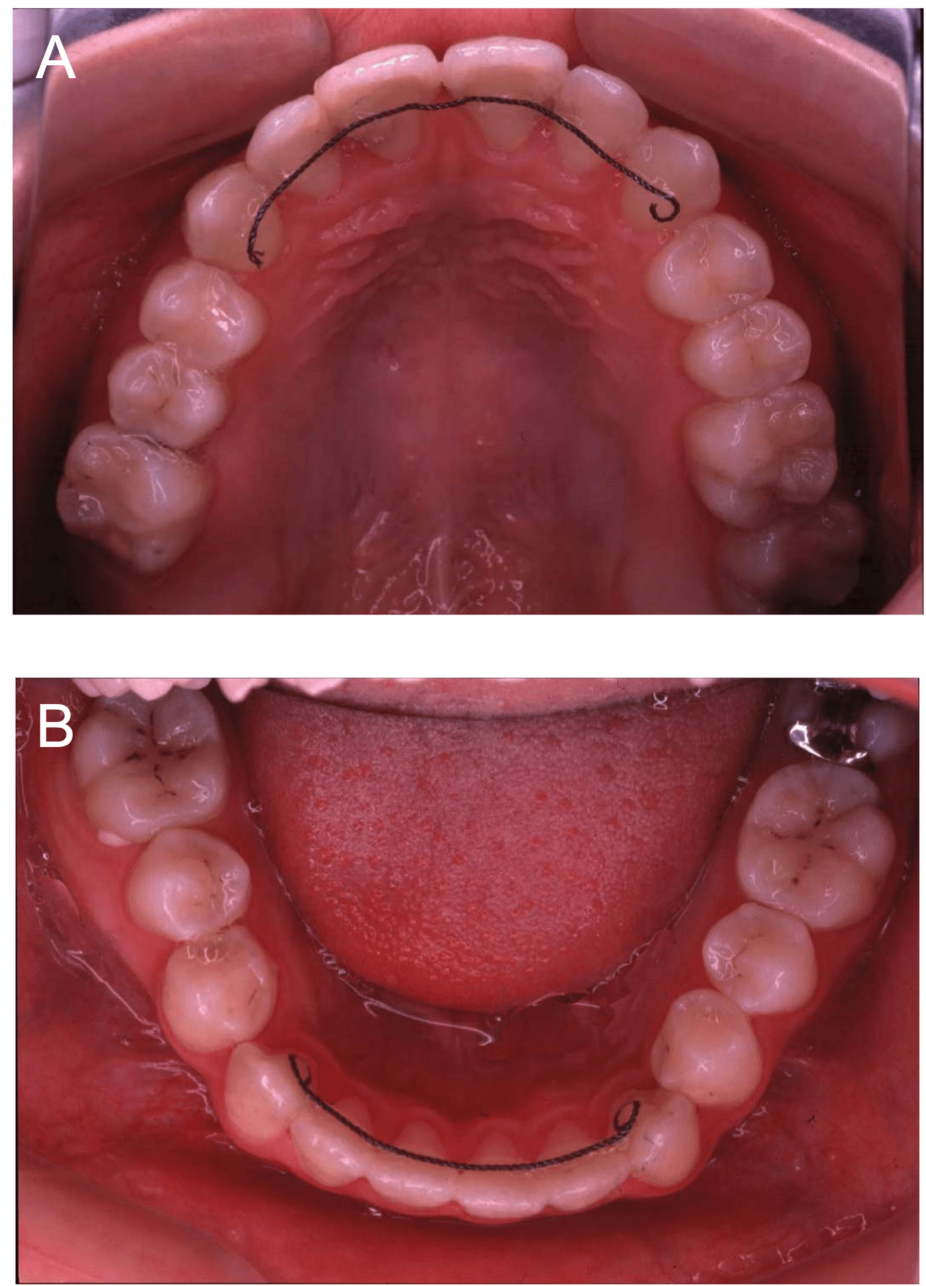
Intraoral photographs at age 19 A: Upper jaw. B Lower jaw. No gross deformities in the upper and lower jaws are observed after the completion of orthodontic treatment

**Figure 15:**
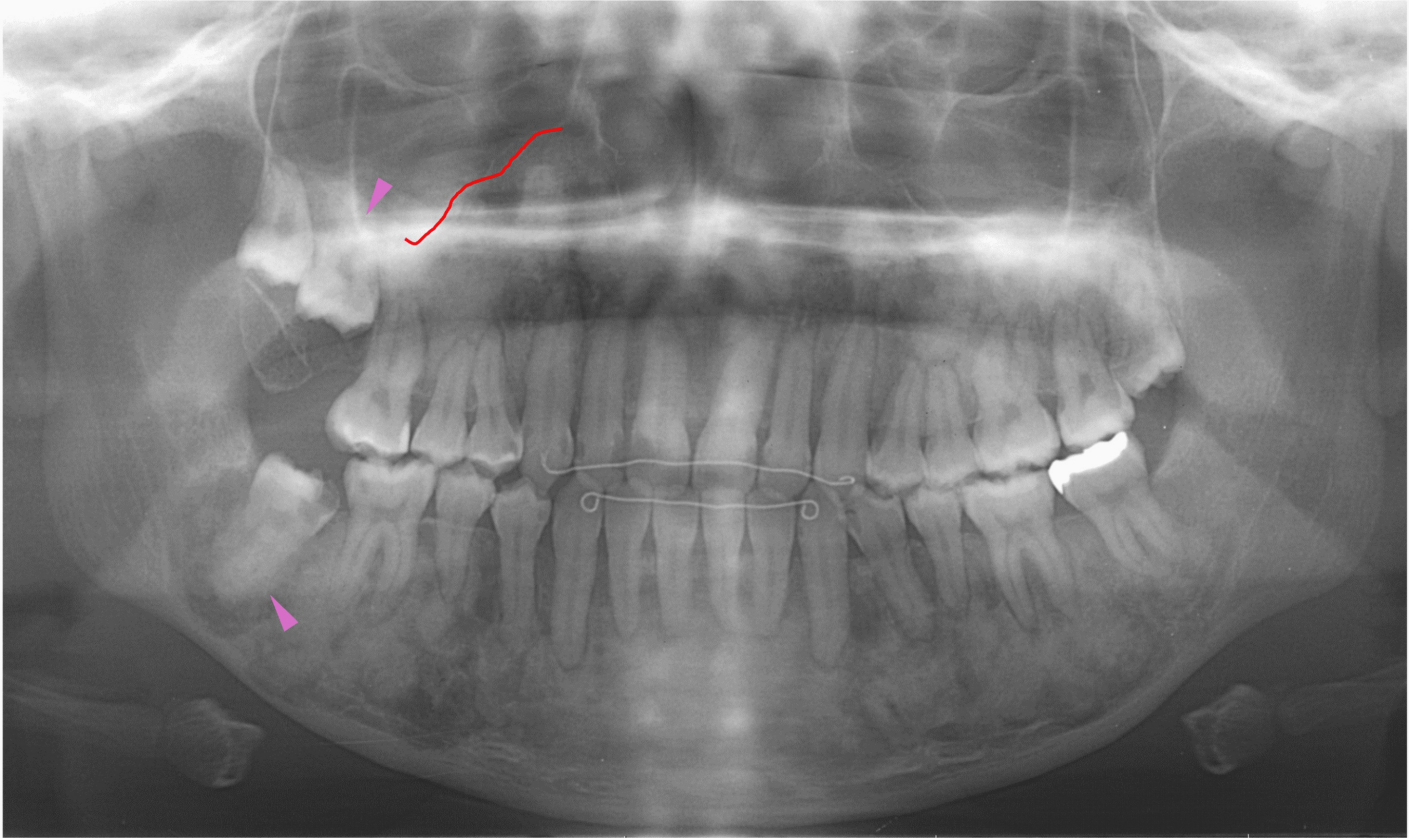
Maxillofacial photograph at age 20 Purple arrowheads indicate osseous adhesions (ankylosis). The red line indicates osseous changes in the right maxillary sinus

**Figure 16:**
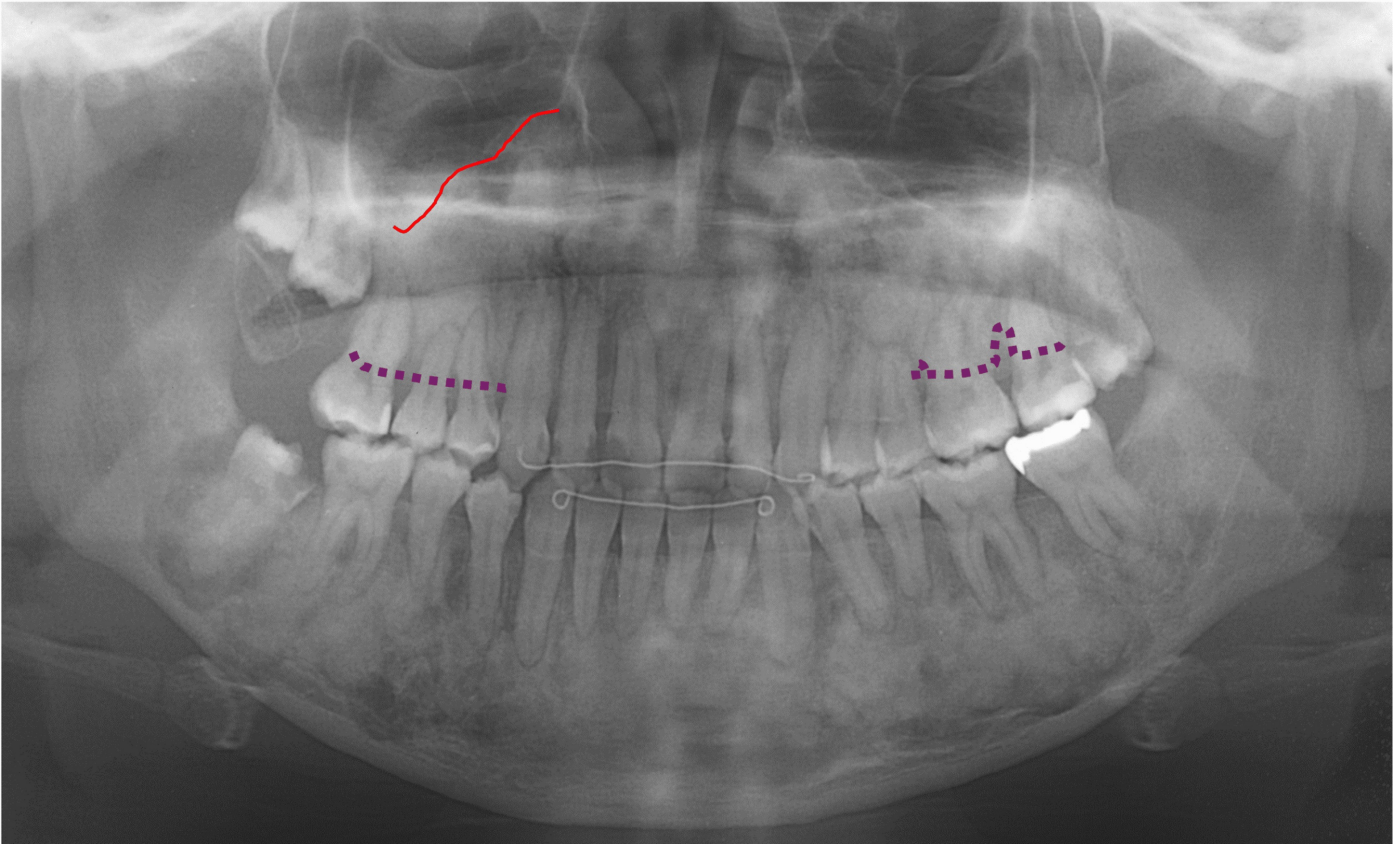
Maxillofacial photograph at age 22 The red line indicates osseous changes in the right maxillary sinus. Purple dotted lines indicate alveolar bone resorption due to periodontitis

Eight months later, at 19 years of age, orthodontic treatment was completed, and no gross deformities in the upper and lower jaws were observed (Figure 14). At 20-22 years of age, radiographic images showed mixed features of bone lesions, such as radiolucent and radiopaque characters (Figures 15, 16). The observed lesions increased in size and appeared to be intensely fused. In the upper and lower molar regions, periodontal ligaments were lost, and osseous adhesions (ankylosis) were observed (Figures 15, 16: purple arrowheads). Osseous changes constrict the right maxillary sinus (Figures 15, 16: red lines). Alveolar bone resorption, possibly due to periodontitis, was also observed in the upper molars (Figure 16, purple dotted lines). These findings indicated that the bone lesions in the jawbones grew further, with the affected areas expanding to almost the whole bone region in the late adolescent stages.

**Figure 17:**
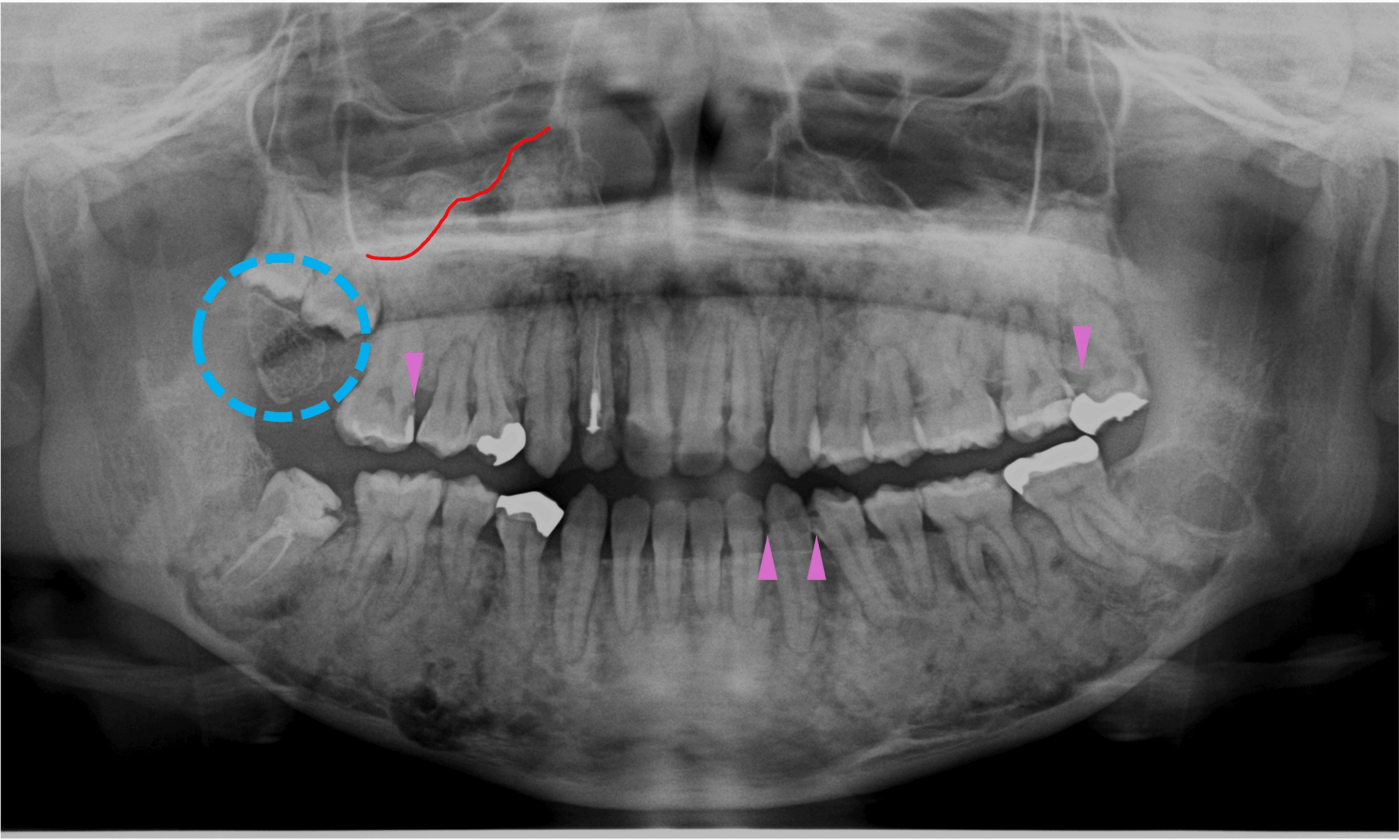
Maxillofacial photograph at age 27 The red line indicates the narrowed right maxillary sinus due to the development of a bone lesion beyond the height of the upper edge of the inferior nasal concha. The blue dotted circle indicates the impacted second and third molars in the upper right jaw. Purple arrowheads indicate multiple carious cavities

**Figure 18:**
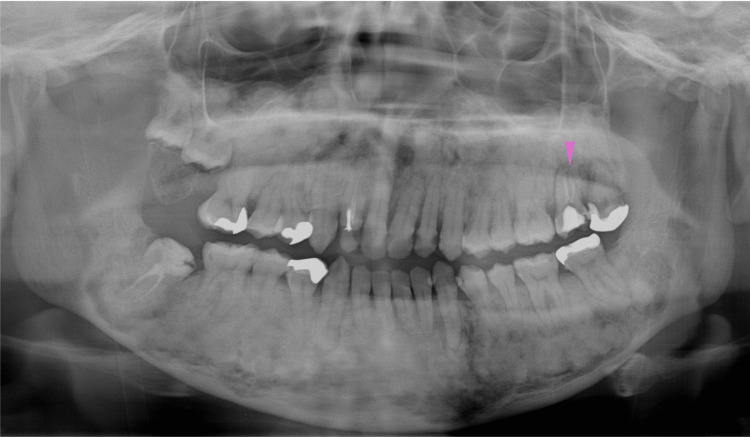
Maxillofacial photograph at age 32 The purple arrowhead indicates performed root canal treatment in the left maxillary second molar

At 27 years of age, the right maxillary sinus appeared to be narrowed because of the development of a bone lesion beyond the height of the upper edge of the inferior nasal concha (Figure 17, red line). In addition, the second and third molars in the upper right jaw were completely impacted, indicating alveolar bone formation covering these teeth (Figure 17, blue dotted lines). Due to pregnancy and subsequent childcare, this patient became less conscious of oral care and developed multiple carious cavities (Figure 17, purple arrowheads). At 32 years of age, owing to the progression of caries, root canal treatment was performed on the left upper second molar (Figure 18, purple arrowhead).

**Figure 19:**
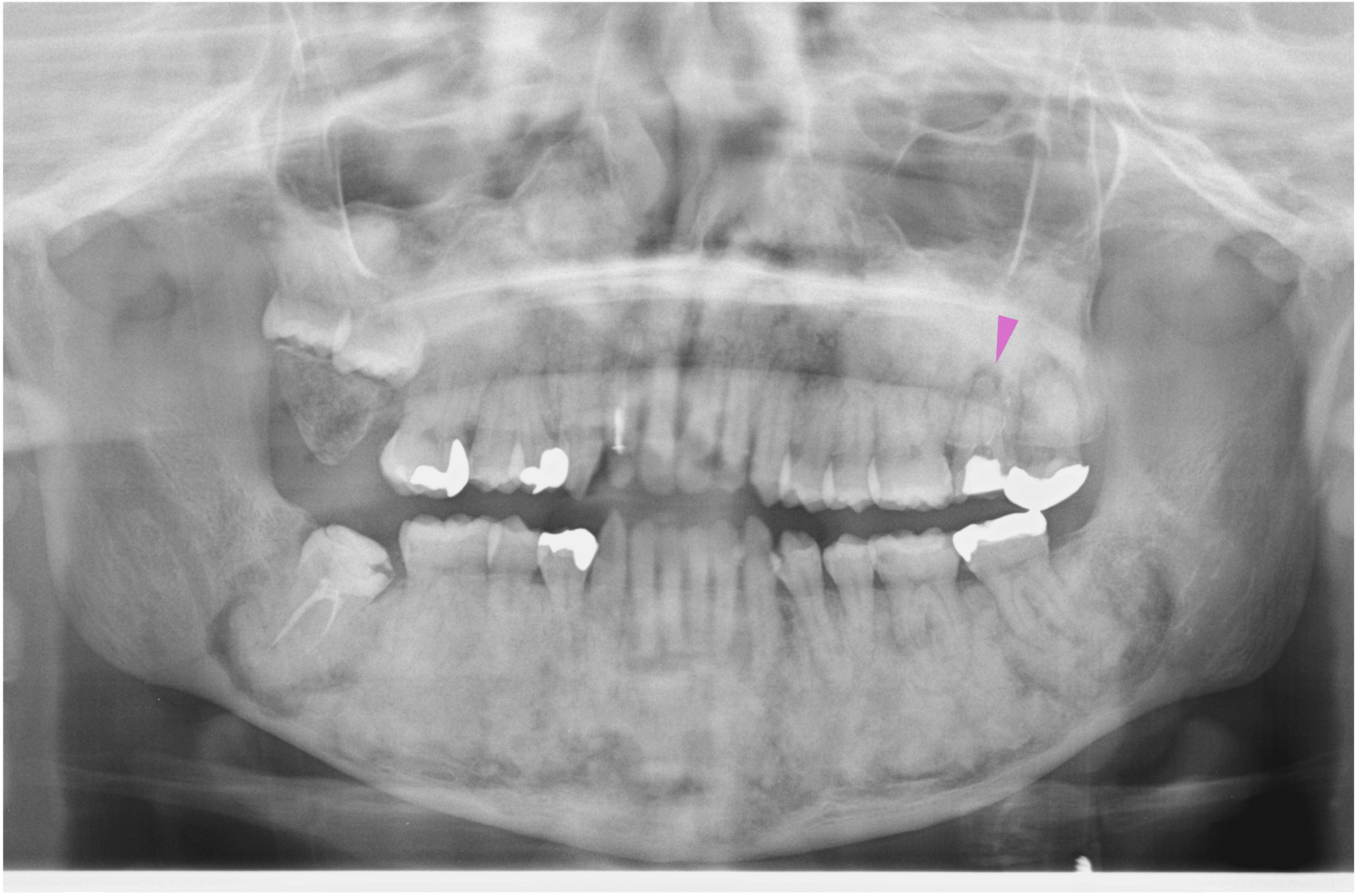
Maxillofacial photograph at age 33 The purple arrowhead indicates a radiolucent area in the apical region of the left maxillary second molar

**Figure 20:**
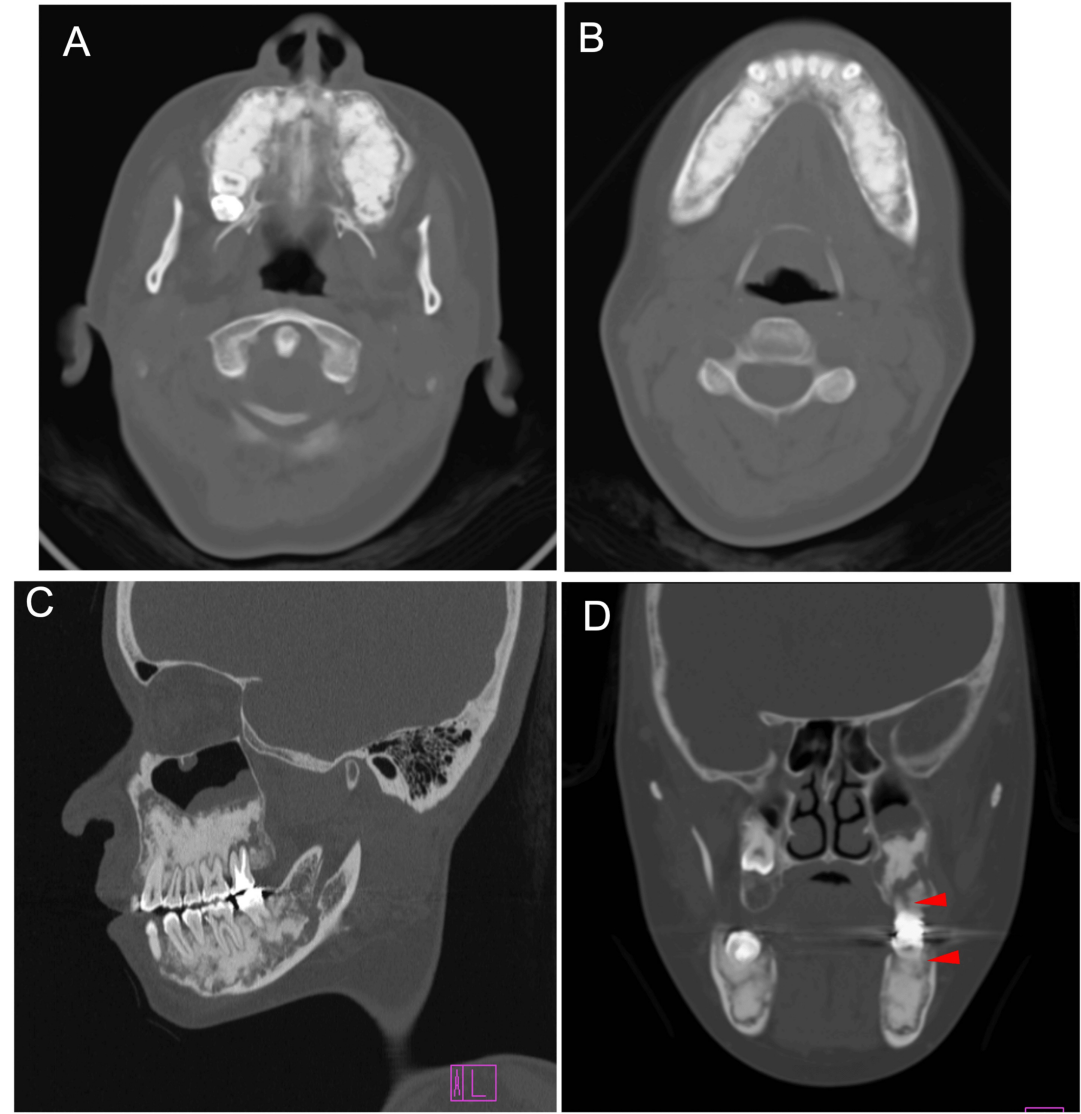
CT images illustrating jawbone lesions at age 33 A: Upper jaw. B: Lower jaw. C: Lateral view. D: Frontal view. Red arrows indicate cortical bone perforation and fistula formation CT: computed tomography

However, at 33 years of age, a radiolucent area reappeared in the apical region of the left maxillary second molar. Chronic apical periodontitis is thought to cause osteomyelitis of the left maxillary bone (Figure 19, purple arrowhead). CT images taken in the same year showed the apparent development of bone sclerosis throughout the maxilla and mandible (Figures 20A, 20B). The right maxillary sinus appeared narrower (Figure 20C). Radiographically, the left maxillary osteomyelitis caused by chronic apical periodontitis of the left maxillary second molar was accompanied by left maxillary sinusitis, which was associated with cortical bone perforation and fistula formation in the same area (Figure 20D, red arrows). Although purulent osteomyelitis of the jaws during adult life is a characteristic feature of GDD, the present case only experienced localized osteomyelitis induced by chronic apical periodontitis in the maxilla.

**Figure 21:**
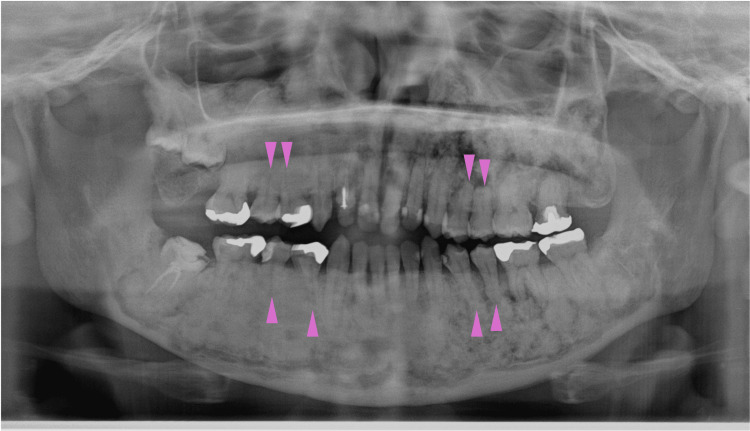
Maxillofacial photograph at age 41 Purple arrowheads indicate shortening of the roots

At 41 years of age, shortening of the roots of the first and second premolars on both sides of the upper and lower jaws was observed (Figure 21, purple arrowheads). The apical regions of the same area showed radiolucency on the left side; however, on the right side, they exhibited sclerotic changes, making it difficult to identify the periodontal ligament, suggesting bone ankylosis at this site. Alveolar bone regeneration after the extraction of the upper left wisdom tooth appeared to be normal.

## Discussion

Jaw lesions observed in the GDD patients fit within the spectrum of “fibro-osseous lesions” that show the replacement of normal bone architecture by benign fibrous tissue composed of fibroblasts and collagen with various amounts of mineralized material [3,7]. The jaw lesions in our case began with multiple lobular or amorphous radiopacities in the vicinity of the tooth germ of the maxilla and mandible at nine years of age, and in mixed dentition (Figure 5). Slight sclerosis of the jawbones has already been recognized before the completion of permanent dentition. Although the jaw lesion is asymptomatic, it gradually enlarges throughout the alveolar process. Some lesions arose from the vicinity of the tooth germ at nine years of age, while others arose from the inside of the radiolucent area adjacent to the root of the tooth at 12 years of age. They then fuse and grow. No histopathological examinations were performed for this patient.

Previous findings of jaw lesions in patients with GDD revealed a pattern similar to that of cemento-osseous dysplasia, showing numerous rounded, calcified, cementum-like structures with fibroblastic stroma [3,8]. This long-term follow-up study of a GDD case revealed that the jawbone lesions showed a progressive pattern similar to that of florid osseous dysplasia [9]. Indeed, at the onset of bone lesion development, we observed focal radiolucent (osteoporotic) areas at the apex of the teeth. These radiolucent areas were expanded and fused, suggesting progression of osteolysis. In the next stages, radio-opaque areas progressively develop from the radiolucent areas, suggesting the formation of cemento-osseous lesions, as previously reported in patients with GDD [3,8]. 

The general radiographic features of this patient also reminded us of McCune-Albright syndrome, which shares similar radiographic findings with GDD [10]. McCune-Albright syndrome is a rare genetic disorder, arises from a somatic activating mutation of a GNAS gene encoding a guanine nucleotide-binding protein G(s) subunit alpha. This syndrome shows fibrous dysplasia in the jawbones, while GDD shows cemento-osseous dysplasia. Fibrous dysplasia on conventional radiography includes a ground-glass appearance, completely radiolucent (cystic) lesions, sclerotic lesions or mixed cystic and sclerotic lesions, well-circumscribed margins (geographic pattern), with or without a sclerotic border, and expanded lesions with a shell that is thick, thin, or showing small perforations and/or endosteal scalloping.

Another characteristic feature of McCune-Albright syndrome is skin pigmentation, called café-au-lait spots. Furthermore, this syndrome is not considered to show autosomal dominant inheritance. Therefore, transplantation of stromal cells obtained from the lesion of a GDD patient into immunocompromised mice showed close mimicry of the native lesion (as observed by histopathological examinations), including the sporadic formation of psammomatoid bodies, suggesting an intrinsic abnormality of bone/cementum-forming cells [3]. This long-term radiographic study demonstrated that cemento-osseous lesions develop with age and even show remodeling of the lesion. 

A case reported by Riminucci et al. presented with bilateral, relatively symmetric, expansile lesions in the maxillary bones associated with a sinus infection at 13 months of age [3]. Otaify et al. reported a case where the patient’s face appeared normal at birth, but by two months of age, progressive enlargement and expansion of the maxilla and mandible were evident [8]. The GDD patient reported in this study did not show functional issues associated with the developing lesions, such as pain and swelling, until adulthood. Therefore, the onset (nine years of age) and symptoms of maxillofacial bone in our case appeared to be milder than other reported cases.

The skeletal features of GDD include bilateral thickening of the diaphyseal cortices of long bones that involves osteopetrosis-like sclerosis, bone fragility, frequent bone fractures at a young age, and bowing of limb bones. Generally, the potential cause of osteopetrosis is the absence of bone resorption ability with the absence or functional loss of osteoclasts, which occasionally accompanies retarded tooth eruption, indicating low turnover of bone metabolism. Riminucci et al. reported a case of GDD with sustained bone fractures, all of which occurred as a result of minimal trauma at the tibia, fibula, clavicles, and wrist [3]. In this case, bone turnover did not show any obvious pathological changes. The Z score of the bone densitometry measurement of the spine using dual-energy X-ray absorptiometry (DXA) was within the normal range. An iliac crest biopsy specimen also showed unremarkable changes in the rate of bone formation and resorption (percentage of trabecular bone volume [BV/TV%]; normal range for age: 13.5-22.9 years). Serum electrolytes, calcium, phosphorus, magnesium, parathyroid hormone, 25- and 1,25-vitamin D, thyroid function tests, and other typical bone markers were normal, although alkaline phosphatase (ALP), a marker of bone formation, was only minimally elevated (345 U/L; normal range: 145-320 U/L).

However, Rolvien et al. reported a case of high bone turnover in a 13-year-old male with GDD who showed recurrent diaphyseal fractures of the femur and a novel de novo missense mutation in the ANO5 gene [11]. The high turnover bone pathology in this case was confirmed through DXA, high-resolution peripheral quantitative computed tomography (HR-pQCT), and serum analyses, as well as the analysis of an iliac crest biopsy specimen, where osteoblast and osteoclast indices were remarkably increased. Otaify et al. reported a case of severe GDD prenatally diagnosed as bilateral femur fractures that was first identified on a 20-week prenatal ultrasound study [8]. This case showed increased serum levels of ALP and Cathepsin K at 15 months of age, indicating high bone turnover. A recent study reported two severe cases of GDD showing a fetal appearance of bone bowing, and one of them also showed an intraoral lesion [12]. In our case, thickness and slight bowing were observed in the diaphyseal cortices of the femurs, tibiae, and ulnae. As our patient did not suffer from any fractures, we did not conduct any orthopedic examinations. Therefore, the skeletal phenotype of our patient appeared to be relatively mild in comparison to that in the reported cases of GDD.

Although the etiology and pathogenesis of cemento-osseous dysplasia remain uncertain, this study clearly illustrates the onset and progression of jaw lesions in patients with GDD. This patient had no symptoms, including bacterial infection or bone fracture, owing to the complete and comprehensive healthcare provided by her parents and herself. Furthermore, lifetime orthopedic follow-up and dental care are mandatory, since the pathological relationship between the skeletal condition of GDD and age-related destructive bone diseases, such as periodontitis, osteoporosis, and osteoarthritis, has not been assessed. 

Owing to orthodontic treatment, the patient’s teeth were aligned, and the patient obtained good occlusal conditions and a good aesthetic profile. Orthodontic appliances can increase the risk of caries and periodontal disease because they can be a plaque retention factor [13]. Orthodontic force affects bone metabolism in the periodontal tissue, which might have affected the progression of cemento-osseous lesions in this patient. Our case proves that orthodontic treatment can be accomplished in patients with GDD without serious problems. Wisdom tooth extraction did not cause any inflammatory lesions. In our case, interventional dental check-ups and professional care were performed thoroughly. It can be emphasized that professional dental care is essential to avoid jawbone infections, causing osteonecrosis of the jawbones, which is often observed in other patients with GDD. 

Patients with GDD have shown variation in the age of onset and the expression of disease phenotypes of the maxillofacial and long bones, even within the same family whose affected members all share the same mutation [3,8,11,12,14-25], which may result from mutations in potential modifier genes, including COL5A1and COL1A1 [15,26,27]. Interestingly, these genes are also related to osteogenesis imperfecta. The Ano5 mutation observed in a GDD patient knock-in mouse (Ano5KI/KI) replicated GDD-like skeletal features [28]. The phenotypes of several Ano5-knock-out mice have been reported; however, the described phenotypes vary even in cases with no obvious phenotype [29-33]. Furthermore, GDD mutations are mainly concentrated within exon 11 of ANO5, causing osteoblastic dysfunction [34-36], while other mutations cause muscular dystrophy, mostly without a bone phenotype [37]. These findings suggest that slight functional changes in the ANO5 protein and associated molecules are involved in GDD. 

This study has some limitations. Though this long-term radiographic analysis of a GDD patient described the onset and development of a jawbone lesion, it is still hard to determine how each new lesion develops and affects surrounding tissues. Quantitative measuring of lesion size or volume change over time could further strengthen our understanding of the developmental aspect of bone lesions.

## Conclusions

Our long-term follow-up study of a patient with the original GDD pedigree demonstrated the onset and development of jawbone lesions. This patient lived her life without serious dental and orthopedic problems, suggesting that the case was milder than some reported in the relevant literature. We emphasize the importance of regular orthopedic follow-up as well as dental care over a lifetime to avoid atypical bone fractures and osteonecrosis of the jawbones. The severity of the GDD phenotypes varies among patients, even within the same family. Further genetic investigation and model studies to understand the pathophysiology are required for a more precise diagnosis, clinical prediction, and better treatment.
